# Quantifying whole bladder biomechanics using the novel pentaplanar reflected image macroscopy system

**DOI:** 10.1007/s10237-023-01727-0

**Published:** 2023-05-30

**Authors:** Grant Hennig, Pragya Saxena, Eli Broemer, Gerald M. Herrera, Sara Roccabianca, Nathan R. Tykocki

**Affiliations:** 1 Department of Pharmacology, University of Vermont Larner College of Medicine, Burlington, VT 05405 USA; 2 Department of Mechanical Engineering, Michigan State University College of Engineering, East Lansing, MI 48824 USA; 3 Department of Pharmacology and Toxicology, Michigan State University College of Osteopathic Medicine, East Lansing, MI 48824 USA

**Keywords:** Compliance, Bladder, Stress, Imaging

## Abstract

Optimal bladder compliance is essential to urinary bladder storage and voiding functions. Calculated as the change in filling volume per change in pressure, bladder compliance is used clinically to characterize changes in bladder wall biomechanical properties that associate with lower urinary tract dysfunction. But because this method calculates compliance without regard to wall structure or wall volume, it gives little insight into the mechanical properties of the bladder wall during filling. Thus, we developed Pentaplanar Reflected Image Macroscopy (PRIM): a novel ex vivo imaging method to accurately calculate bladder wall stress and stretch in real time during bladder filling. The PRIM system simultaneously records intravesical pressure, infused volume, and an image of the bladder in five distinct visual planes. Wall thickness and volume were then measured and used to calculate stress and stretch during filling. As predicted, wall stress was nonlinear; only when intravesical pressure exceeded ~ 15 mmHg did bladder wall stress rapidly increase with respect to stretch. This method of calculating compliance as stress *vs* stretch also showed that the mechanical properties of the bladder wall remain similar in bladders of varying capacity. This study demonstrates how wall tension, stress and stretch can be measured, quantified, and used to accurately define bladder wall biomechanics in terms of actual material properties and not pressure/volume changes. This method is especially useful for determining how changes in bladder biomechanics are altered in pathologies where profound bladder wall remodeling occurs, such as diabetes and spinal cord injury.

**Supplementary Information:**

The online version contains supplementary material available at 10.1007/s10237-023-01727-0.

## Introduction

Bladder compliance is measured clinically and experimentally as the change in volume for a given change in pressure during bladder filling, due in part to the inability to reliably calculate bladder wall volume without ultrasound imaging (Dorr 1992; Nagle et al. 2017). Unfortunately, measuring compliance in this manner erroneously makes two key assumptions. First, it assumes that all healthy bladder walls are composed of cells and matrix components in similar proportions and thus have a similar elastic modulus. Second, it assumes that all healthy bladders have comparable total wall volume and gross geometry. These two assumptions mean that measuring compliance as ΔV/ΔP is imprecise and easily skewed by the size and thickness of the bladder wall. For example: a large bladder with a thin, stiff wall could appear highly compliant if not filled to the point of engaging viscoelastic elements of the wall. One could then conclude erroneously that the bladder is extremely compliant when it is indeed not. These assumptions and omissions have resulted in immense variability in bladder compliance even in healthy individuals and made it extremely difficult to deduce “normal” compliance (Wyndaele et al. 2011).

When considering the entirety of filling, the mechanical behavior of the bladder wall is nonlinear. Intravesical pressure changes very little for very large filling volumes but increases exponentially thereafter (Parekh et al. 2010). Measures of clinical compliance, however, are derived only from the low end of total bladder capacity (Wyndaele et al. 2011) and thus do not give a complete picture of the mechanical properties of the bladder wall. By measuring pressure and bladder geometry over the entirety of filling, we could calculate the mechanical parameters of the bladder wall in the same way as mechanical engineers would measure the properties of any pressure vessel. This idea has been applied to human subjects by using ultrasound to take sagittal and transverse images (1 per minute) during cystometry (Nagle et al. 2017). However, the high cost and low resolution of this technique makes it extremely difficult to implement in animal models and still only gives a partial view of bladder geometry during filling. In order to calculate the mechanical properties of the bladder wall, new methods are required by which bladder wall geometry and intravesical pressure can be measured inexpensively and in real time during filling.

Almost all biological imaging techniques have one major flaw: by their very nature, the images they collect only contain two-dimensional information for a given point in time. To overcome this limitation, advanced confocal and light-sheet microscopy techniques were developed that take sequential images through the *z* plane of a sample to reconstruct tissue in three dimensions (Bouchard et al. 2015; Keller and Ahrens 2015). However, even the fastest coded light-sheet array microscopes can only reach speeds of 6–10 volumes per second in very small, optically cleared tissue sections and with an absolute minimum amount of movement (Ren et al. 2020). In order to image live tissue in three dimensions and at speeds greater than 10 Hz, new techniques must be developed. Thus, we invented “Pentaplanar Reflected Image Macroscopy” (PRIM), a new technique where a single camera and four fixed mirrors are used to record five imaging planes of a sample in a single camera frame. Because the camera, optics, light source, and the sample are stationary, all focal planes are taken at once and at high speeds (75 Hz or more, depending on the camera). Because the PRIM System allows rapid and accurate measurements of bladder shape, internal volume, and wall volume over the entirety of bladder filling while also capturing frame-locked intravesical pressure recordings, compliance can instead be reported as Cauchy stress/stretch instead of ΔV/ΔP. Because this equipment is scalable, inexpensive, and easy to use, it could potentially revolutionize imaging of any organ where dynamic movements occur in three dimensions simultaneously; this includes propagation of peristaltic movement in the gut, motility in the stomach or uterus, or even nutation of plant stems during growth.

In this study, we implement the PRIM System to measure bladder wall geometry and pressure and use this information to calculate bladder wall stress and stretch during ex vivo filling of normal mouse bladders. Our measurements were reproducible, robust, and consistent with those made using biaxial mechanical testing of bladder wall samples and other previously published measures of bladder biomechanics (Gloeckner et al. 2002; Nagatomi et al. 2004). Thus, the PRIM System can bring new insight into intact bladder wall biomechanics during filling.

## Methods

### Animal care and use

All animal procedures followed institutional guidelines and were approved by the Institutional Animal Care and Use Committees of Michigan State University (NIH Assurance D16-0054). Male C57Bl/6 mice (9–12 weeks old; Jackson Laboratory, Bar Harbor, ME) were group housed in a temperature- and humidity-controlled environment with a 12 h light/dark cycle. Mice were provided *ad libidum* access to standard chow and water. Mice were euthanized by injection of pentobarbital (> 150 mg/kg i.p.) followed by decapitation.

### Pentaplanar reflected image macroscopy (PRIM) system

**Fig. 1:**
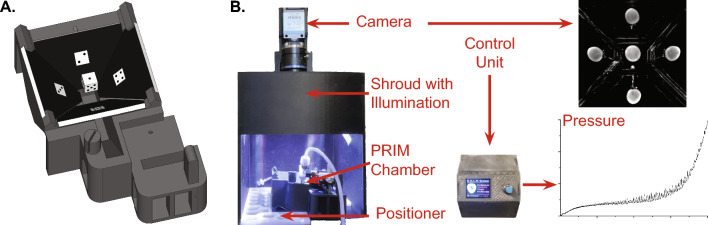
The Pentaplanar reflected image macroscopy (PRIM) system. **A** Rendering of the PRIM chamber and mirrors, illustrating each of the five captured visual planes. **B** The entire system consists of the chamber, shroud, positioner, camera, and control unit. In addition to video images, other data (e.g., pressure) can be recorded. Time-linked pressure and imaging data can be combined to calculate the mechanical properties of the sample over time (e.g., Cauchy stress versus stretch of a mouse urinary bladder during filling)

The PRIM System consists of four main sections: the PRIM imaging chamber, a digital camera with television lens, the lighting shroud, and the integrated control unit (Fig. 1A, B). Unless otherwise specified, all custom-designed pieces were three-dimensional printed from PETG plastic using a fused deposition modeling printer (Prusa i3 Mk3S; Prusa Research, Czech Republic). The PRIM chamber itself consists of a custom-designed ABS imaging chamber, four mirrors, the mounting cannula, and chamber positioner. The chamber is filled with liquid through a tube that enters in the center, below the sample. Fluid level is maintained using a peristaltic pump and suction tube (Warner Instruments, USA). Back-coated mirrors (3.18 mm thickness; The Home Depot, USA) were cut and shaped in house. Mirrors are fixed in position at 45° angles relative to the central specimen, which is placed on the cannula for ex vivo filling using a standard syringe pump. The monochrome digital camera (DMK33UX250, 5 megapixel, 3.45 µm^2^ pixel size; The Imaging Source, USA) and television lens (TCL1616, 16 mm focal length, F1.6–16; The Imaging Source) were mounted atop the lighting shroud roughly 12 cm above the imaging chamber on a custom camera mount that allowed for focusing and centering of the image. The camera sat atop the shroud, which was sized to allow full illumination of the sample without inclusion of the lights themselves in the reflected image. The integrated control unit consisted of an Arduino microcontroller, Wheatstone bridge amplifier shield (Robotshop.com), PicoBuck LED driver (COM-13705, Sparkfun), in-line pressure transducer (DYNJTRANSMF, Medline) and 12 V DC power supply. As designed and assembled, the integrated control unit had a pressure resolution of 0.07 mmHg that remained linear across a range from 0 to 40 mmHg. Image pixel size was 64 µm^2^. Pressure transducers were calibrated using a sphygmomanometer. Pixel size was confirmed in every image using the known width of the bottom of right-hand mirror, as measured by vernier caliper. Both pressure readings and camera triggering were regulated by the Arduino microcontroller to ensure a single pressure reading was matched with each image frame, and readings were collected at 10 Hz. Pressure data were collected using *VasoTracker-DataTracker* software (courtesy Dr. Calum Wilson, University of Strathclyde; UK) for later analysis. Video images were collected using *IC Capture* software (The Imaging Source; USA). When properly aligned, five visual planes are recorded in a single image and each image frame correlates to a single pressure and infused volume reading. These data are then used for measurement and analysis of bladder geometry and bladder compliance.

### Ex vivo bladder filling

Urinary bladders were isolated and cannulated using a modified version of our previously described protocol (Heppner et al. 2016). Briefly, the urinary bladder with ureters and urethra was removed and placed in ice-cold Ca^2+^-free HEPES-buffered physiological saline solution (HB-PSS) containing [mM]: NaCl [134], KCl [6], MgCl2 [1.2], HEPES [10] and glucose [7]; pH = 7.4. The bladder and urethra were cleaned of fat and connective tissue and ureters were tied proximal to the bladder wall with 4–0 suture. The tissue was then moved to the PRIM chamber (also containing Ca^2+^-free HB-PSS), cannulated, and affixed with 4–0 suture. HB-PSS was then exchanged for bicarbonate-buffered PSS containing [mM]: NaCl [119], NaHCO_3_ [24], KCl [4.7], KH_2_PO_4_ [1.2], MgCl_2_ [1.2], and CaCl_2_ [2]. PSS buffer was bubbled externally with 5/20/75% CO_2_/O_2_/N_2_ to maintain pH, recirculated by peristaltic pump, and heated to 37 °C using an in-line heater (Warner Instruments). Ca^2+^-replete PSS was infused into the bladder at a constant rate (30 µl/min) through the PRIM chamber’s cannula using a calibrated syringe pump. Ex vivo filling commenced simultaneously with video and pressure recording. To increase the contrast between the lumen and bladder wall, infused buffer was mixed with dark food coloring. Bladders were filled until a maximum pressure of 20–25 mmHg was reached, at which time the infusion and video/pressure recordings were simultaneously stopped. Bladders were then emptied and allowed to re-equilibrate for 5–10 min before the next fill. To simulate preconditioning, fill-empty cycles were repeated at least 5 times for each bladder; the final fill-empty cycle was used for all calculations.

### Bladder wall thickness, volume, and residual estimation

The urinary bladder was assumed to have an elastic wall of constant volume and uniform thickness, which deforms during filling and behaves as a nonlinear expanding vessel (Damaser and Lehman 1995). Here and in the following, lowercase letters will refer to the geometry of the bladder when full (deformed configuration at 20 mmHg) and uppercase letters will refer to the geometry of the bladder when empty (configuration at ~ 0 mmHg).

**Fig. 2:**
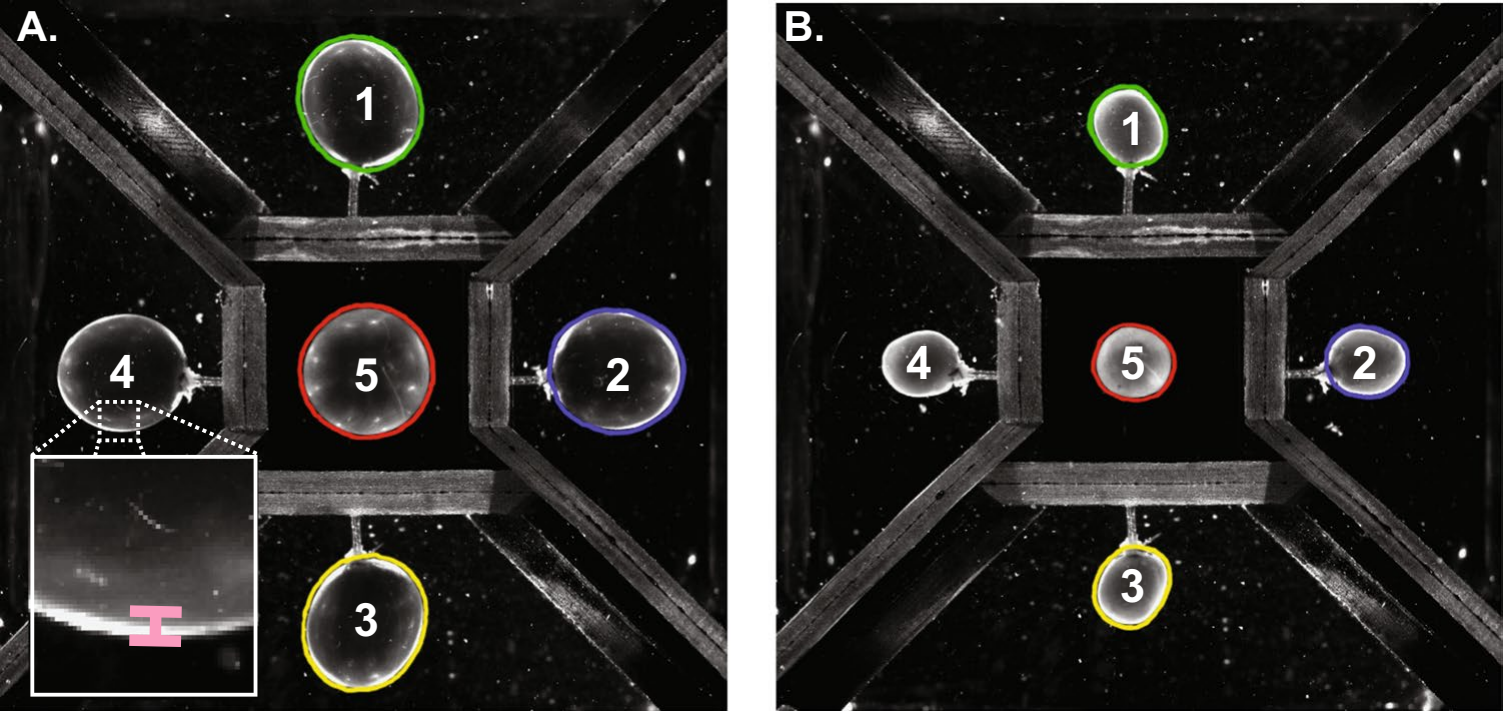
Measurement of planar areas and wall thickness from PRIM images of a full and empty urinary bladder. **A** Starting with the full bladder image, perimeters of each section were drawn and the enclosed area was calculated. Wall thickness was estimated as the area of highest contrast at the bladder edge (inset). **B** The area of each section was again calculated from the empty bladder image as filling began

To measure all dimensions required to calculate the wall volume, the first and last frames of the filling video were extracted and areas of each image plane of the bladder was measured using ImageJ software (NIH) (Fig. 2). Perimeters were manually selected by the contrast between black background and white tissue. The average area of Sects. 1, 2, 3, 4 (representing vertical planes of the bladder) and the area of Sect. 5 (representing a plane perpendicular to the other sections) were first measured. These measurements were collected from images at 20 mmHg (Fig. 2A) and 0 mmHg (Fig. 2B). Bladder wall thickness in the full bladder ($t$) was first estimated by measuring the width of the brightest section of the edge of the bladder wall in the last frame of the filling video (Fig. 2A, inset). Calculated wall thickness was 107.83 ± 19.11 µm (*N* = 6) at 20 mmHg.

**Fig. 3:**
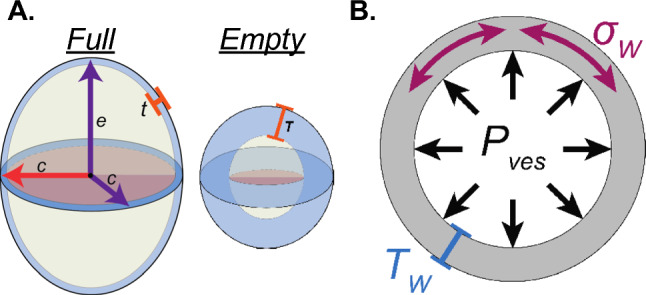
Modeling urinary bladder internal volume, external volume, and wall volume for Cauchy stress calculations. (**A**, left) The internal and external volumes of the full bladder were modeled as ellipsoidal shells with thickness t, comprised of an intersecting circle (pink) of radius c (red) and an ellipse (yellow) with radii c and e (purple). The circle was equal in area to the area of Sect. 5 (See Fig. 2). The ellipse was equal in area to the average area of Sects. 1, 2, 3, 4. Radii of internal volume were calculated by subtracting wall thickness. The difference between these two volumes was the volume of the bladder wall itself (blue). (**A**, right) Because wall volume is conserved, external volume of the empty bladder and the volume of the bladder wall were used to derive wall thickness when empty (T). **B** Wall stress (*σ*W) was calculated by considering the bladder to be a thick-walled (TW) isochoric sphere that undergoes isotropic deformation as intravesical pressure (Pves) increases as the bladder fills

The bladder was next modeled as an ellipsoidal vessel. The area of Sect. 5 was modeled as a circle of equivalent area with radius ${r}_{\mathrm{c}}$. The average area of Sects. 1, 2, 3, 4 was then modeled as an ellipse of equivalent area with radii ${r}_{\mathrm{c}}$ and ${r}_{\mathrm{e}}$.The hollow ellipsoid model was formed by the intersection of the circle with radius ${r}_{\mathrm{c}}$ and an ellipse with radii ${r}_{c}$ and ${r}_{e}$ (Fig. 3A).

The outer volume of the full bladder (${v}_{\mathrm{o}}$) was calculated as the volume of an ellipsoid with radii ${r}_{\mathrm{c}}$ and ${r}_{\mathrm{e}}$:

1 $$v_{{\text{o}}} = 4/3\pi r_{{\text{c}}}^{2} r_{{\text{e}}}$$

Inner full bladder volume (${v}_{i}$) was calculated as the volume of an ellipsoid with radii less wall thickness, as*:*

2 $${v}_{\mathrm{i}}= \frac{4}{3}\pi {{(r}_{\mathrm{c}}-t)}^{2}({r}_{\mathrm{e}}-t)$$

Thus, bladder wall volume (${v}_{\mathrm{w}}$) was the difference between outer and inner bladder volumes, calculated as:

3 $${v}_{\mathrm{w}}={v}_{\mathrm{o}}-{v}_{\mathrm{i}}$$

Residual volume (${v}_{\mathrm{res}}$) was also calculated as the difference between inner volume of the full bladder and infused volume (${v}_{\mathrm{inf}}$):

4 $${v}_{\mathrm{res}}={v}_{\mathrm{i}}-{v}_{\mathrm{inf}}$$

Note that the value of ${v}_{\mathrm{res}}$ is constant throughout the test as well as the analysis.

This procedure could not be replicated to estimate the wall thickness in the empty bladder due to the opacity of the tissue. For this reason, wall thickness in an empty bladder was estimated by assuming that the bladder was undergoing isochoric deformation (Ajalloueian et al. 2018; Damaser and Lehman 1995; Nagle et al. 2017; Roccabianca and Bush 2016). Thus, bladder wall thickness when empty ($T$) is again calculated using Eqs. 1 and 2. Circular (${R}_{\mathrm{c}}$) and elliptical (${R}_{\mathrm{e}}$) radii of the empty (0 mmHg) bladder are derived from Sects. 1, 2, 3, 4, 5 of PRIM images immediately prior to the start of filling (Fig. 2B). Outer bladder wall volume when empty (${V}_{\mathrm{o}}$) was calculated as:

5 $${V}_{\mathrm{o}}= \frac{4}{3}\pi {{R}_{c}}^{2}{R}_{\mathrm{e}}$$

Due to the hypothesis of isochoric deformation, the volume of the bladder wall must be conserved throughout pressurization; therefore, ${v}_{\mathrm{wall}}={V}_{\mathrm{wall}}$. The inner bladder volume can be calculated as:

6 $${V}_{\mathrm{o}}-{v}_{\mathrm{wall}}=\frac{4}{3}\pi {{(R}_{\mathrm{c}}-T)}^{2}({R}_{\mathrm{e}}-T)$$

This allows us to calculate the thickness of the empty bladder $t$ as the real, positive solution to the third-order quadratic equation:

7 $${T}^{3}+\left(-2{R}_{\mathrm{c}}-{R}_{\mathrm{e}}\right){T}^{2}+\left({{R}_{\mathrm{c}}}^{2}+2{R}_{\mathrm{c}}{R}_{\mathrm{e}}\right)T-{{R}_{\mathrm{c}}}^{2}{R}_{\mathrm{e}}+\frac{3}{4\pi }\left({V}_{\mathrm{o}}-{v}_{\mathrm{wall}}\right)=0$$

**Fig. 4:**
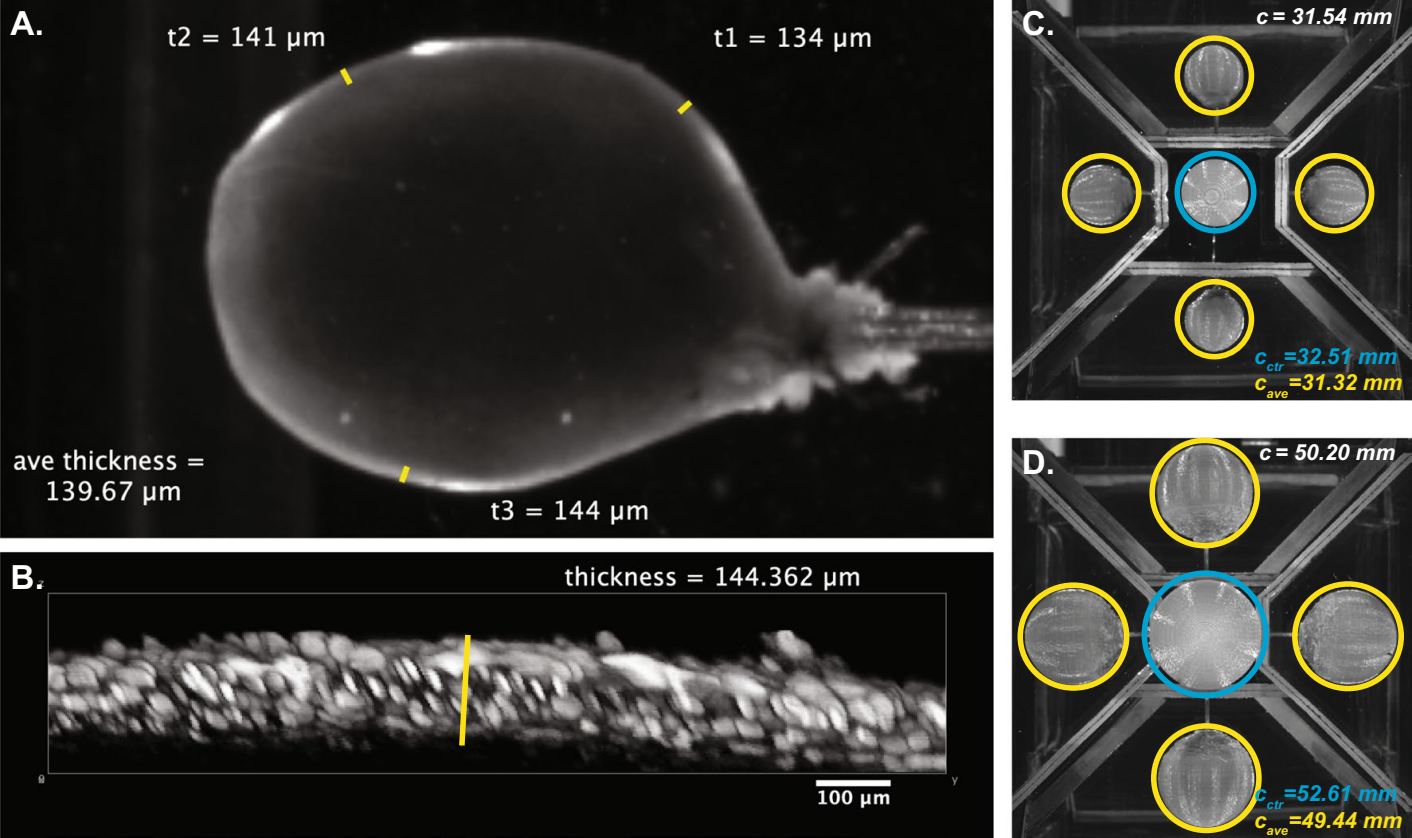
Validation of thickness measurements. **A** Representative PRIM image of a full bladder (25 mmHg). Wall thickness was measured as described at three places across the bladder wall. **B** 3D reconstruction of confocal Z stacks of the same bladder, again at 25 mmHg. **C** and **D** PRIM images of 3D-printed spheres with diameter of 10 mm (**C**) or 16 mm (**D**). Actual (white) and calculated (yellow/cyan) circumferences are noted in each image

As a means of validating the wall thickness measurements, the residual volume when empty (${V}_{\mathrm{res}}$) was calculated as above by subtracting ${V}_{\mathrm{o}}$ from ${v}_{\mathrm{wall}}$ and comparing this value to ${v}_{\mathrm{res}}$ calculated in Eq. 4. If these values were within ± 10% of one another, we considered the wall thickness measurements valid. Two bladders were also used to validate thickness measurements using confocal microscopy (Fig. 4). After recording in the PRIM system to a maximum pressure of 25 mmHg, the bladder was emptied, removed from the cannula, and incubated with 10 µM wortmannin and 5 µM Calcein stain (Thermo Fisher, USA). The bladder was then cannulated again and Z stack images (1 µm thickness) were recorded using confocal microscopy. Error in bladder thickness was 1.57% and 3.25%, respectively.

The accuracy of our measurement methodology and mirror alignment was tested using two model spheres (10 mm diameter and 16 mm diameter). Spheres were placed on the cannula and imaged in both the presence and absence of buffer in the PRIM chamber. Dimensions were then calculated using the above methodology and compared to the known spherical volume. In the absence of buffer, the error in calculated volume was 3.43% and 3.38% for the 10 mm and 16 mm spheres, respectively. In the presence of liquid, the error in calculated volume was 1.41% and 0.98% for the 10 mm and 16 mm spheres, respectively.

### Spherical Cauchy stress calculation

Mechanical forces acting on the bladder wall were calculated by first modeling the bladder to be a three-dimensional pressurized spherical vessel made of elastic material (Fig. 3B), similar to previous descriptions (Nagle et al. 2017). Radius ($\rho$) was calculated for any given value of pressure by modeling the bladder as a sphere equal in inner volume to the sum of the residual volume (${v}_{\mathrm{res}}$) and infused volume (${V}_{\mathrm{inf}}$) at each value of pressure. Thus, the radius ($\rho$) was calculated as:

8 $$\rho =\sqrt[3]{\frac{3\left({V}_{\mathrm{inf}}+{V}_{\mathrm{res}}\right)}{4\pi }}$$

Stretch ($\lambda$) was used as a measure of deformation and was calculated as the ratio of the current radius by the undeformed (empty) radius:

9 $$\lambda =\rho /R$$

Lastly, we used the measurements derived above to calculate Cauchy stress during bladder filling:

10 $${\sigma }_{W}=\frac{{p}_{\mathrm{ves}}\bullet \rho }{2\bullet {t}^{^{\prime}}}$$

where ${p}_{\mathrm{ves}}$ is intravesical pressure, $\rho$ is current spherical radius, and is $t^{\prime } = T/\lambda^{2}$ wall thickness in the current configuration and how it relates to the initial thickness ($T$) due to the assumption of isochoricity. We assume that the thickness in the reference configuration is equal to that calculated using Eq. (7). Stress and stretch values were then plotted for each time point during ex vivo bladder filling.

### Biomechanical metrics

These data were also used to derive biomechanical metrics, as described previously (Tuttle et al. 2022; Zwaans et al. 2022). Briefly, we define *E*_low_ and* E*_high_ as the slope of the spherical stress-stretch curve for low and high values of pressure, respectively. We identified these values as the slope of the linear fitting corresponding to the highest *R*^2^, when fitting the first (*E*_low_) or last (*E*_high_) n points of the curves (where 5 $\le$ *n* $\le$ *n*_tot_). We also evaluated linearized stiffnesses as the slope of the stress-stretch curve for three physiologically relevant values of pressure: in vivo filling (5 mmHg), initiation of voiding (10 mmHg), and maximum filling (20 mmHg). The corresponding linearized stiffnesses (*E*_5_, *E*_10_, and *E*_20_) were calculated as follows: first, for each experimental dataset, we identified the specific pressure ± 2 mmHg, as well as the corresponding filling volumes (except for maximum filling pressure, where we considered the only the lower boundary of the interval). Then, we calculated the values of spherical stress and stretch using Eqns. (9, 10) and, finally, we performed a linear fitting. The slope of the linear fitting represented the corresponding linearized stiffness.

### Statistics

Statistical significance between groups was established using a two-tailed, paired Student's *t* test (*α* = 0.05). One-way ANOVA was used to determine if the means of three independent outcomes were significant when compared (*α* = 0.05) followed by Tukey’s post hoc analysis to compare individual means. Calculations were performed using Microsoft Excel or GraphPad Prism 9.5 (GraphPad Software, San Diego, CA). Values are expressed as means ± SEM. Differences with *P* values < 0.05 were considered statistically significant**.**

## Results

### Pressure–volume relationships

**Fig. 5:**
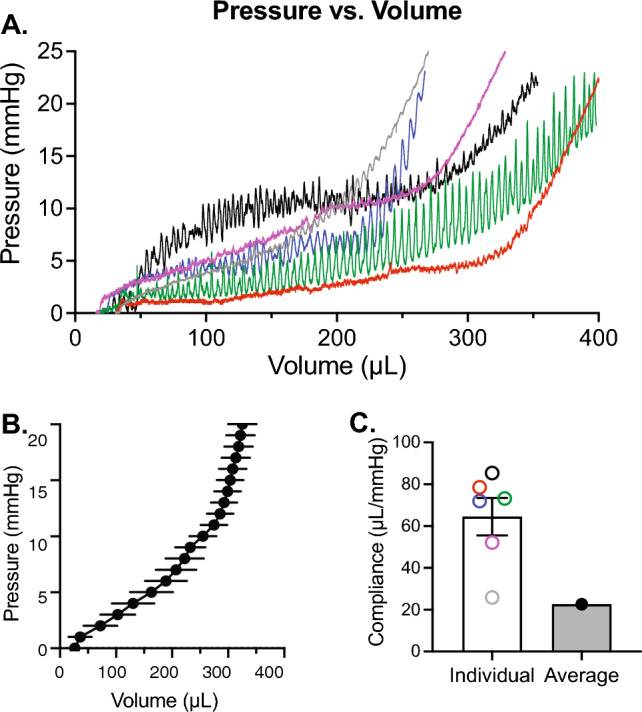
Pressure vs. volume comparisons between bladders is skewed by bladder wall geometry. **A** Graphs of pressure vs. volume for a group of male mouse urinary bladders of similar ages. **B** Summary graphs showing average pressure vs. volume. **C** Compliance measured in this way cannot distinguish between geometric stiffness and material stiffness; thus the compliance of the average curve appears almost 3 times stiffer than the mean of the individual curves. Circle color matches the trace from which it was calculated. Data shown as mean ± SEM. *N* = 6

Generally, bladder filling consists of four phases, three of which are present in ex vivo bladder experiments (Chancellor 2016; Heppner et al. 2016; Tykocki et al. 2019). An initial increase in pressure is then followed by a sustained period, during which pressure increases minimally as intravesical volume increases (“filling phase”). This is then followed by a rapid increase in pressure as maximum capacity was reached. Using C57Bl/6 mouse bladders, we measured the pressure–volume relationship during ex vivo filling (Fig. 5). Each trace had a tri-phasic shape typical of ex vivo bladder filling (Fig. 5A). The pressures and volumes at which these phases occurred, however, varied substantially between bladders. This distorted the average pressure–volume relationship of these experiments (Fig. 5B). “Compliance” is also often considered clinically as a measure of bladder function, wherein the change in volume per change in pressure is noted during filling (Chancellor 2016). Because measures of pressure and volume alone cannot distinguish between geometric stiffness mechanical stiffness, differences in tissue composition, bladder size, or bladder wall thickness skew compliance calculations and are thus not representative of the actual mechanical properties of the bladder wall (Fig. 5C).

### Stress-stretch relationships

**Fig. 6:**
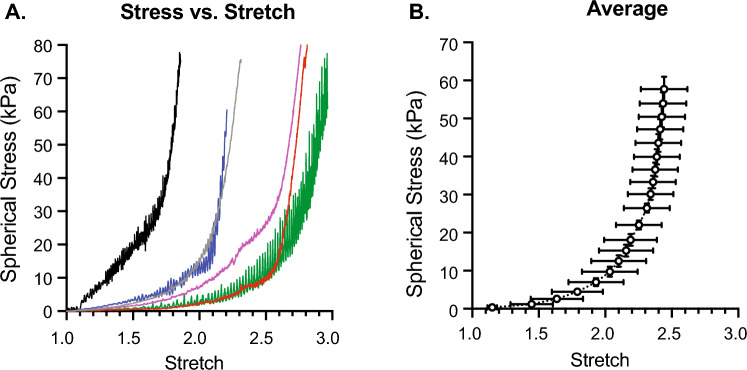
The stress vs. stretch relationship accurately models the biomechanical properties of the bladder wall during all stages of filling by accounting for bladder wall geometry. (A) Graphs of stress vs. stretch for the same group of male mouse urinary bladders as used in Fig. 5. (B) Summary graphs showing average stress vs. stretch at each whole pressure value (1–20 mmHg). Bladders with the most spherical stress per stretch have the lowest compliance. Data are shown as mean ± SEM. N = 6

Using the PRIM System, we were able to use bladder geometry, infused volume, and intravesical pressure to measure the biomechanical characteristics of the bladder wall in terms of spherical stress *versus* stretch (Fig. 6; Supplemental Video 1). Because changes in initial geometry and thickness of the bladder are included in calculations of stress and stretch, the mechanical properties of the bladder wall can be calculated and compared at any time during filling. Differences in compliance are indicated by rightward shifts in the stress-stretch curve. Thus, the least compliant bladder is shown in black while the most compliant bladder is in green.

### Comparing compliance measurements from pressure–volume vs. stress-stretch

**Fig. 7:**
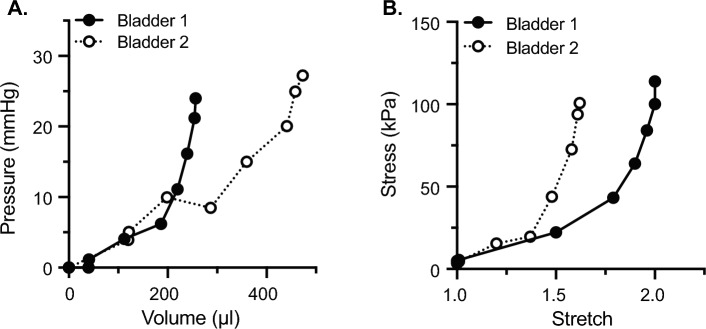
Pressure vs. volume curves can lead to false conclusions regarding bladder wall mechanical stiffness. Pressure–volume **A** and stress-stretch **B** curves for the same two bladders. After accounting for wall volume, bladder 1 is actually more compliant than Bladder 2

To exemplify the benefits of including bladder geometry when measuring bladder wall biomechanics, Fig. 7 presents a data set where different conclusions regarding bladder wall compliance can be made when considering clinical compliance vs. mechanical compliance. In Fig. 7A, clinical compliance (ΔV/ΔP) is measured as 28.83 µL/mmHg for bladder 1 *versus* 48.25 µL/mmHg for bladder 2. Thus, the conclusion would be that bladder 2 was more compliant than bladder 1 given the greater change in volume per increase in pressure. If geometric characteristics of the bladder are used to calculate mechanical stress and stretch during filling, it becomes apparent that bladder 2 is far more distensible than bladder 1. So, while the capacity of Bladder 2 is larger than Bladder 1, its wall is actually significantly stiffer. Thus, calculating bladder compliance in terms of stress and stretch considers not only bladder capacity, but also the thickness and makeup of the wall itself.

### Modeling bladder wall biomechanics from PRIM data

**Fig. 8:**
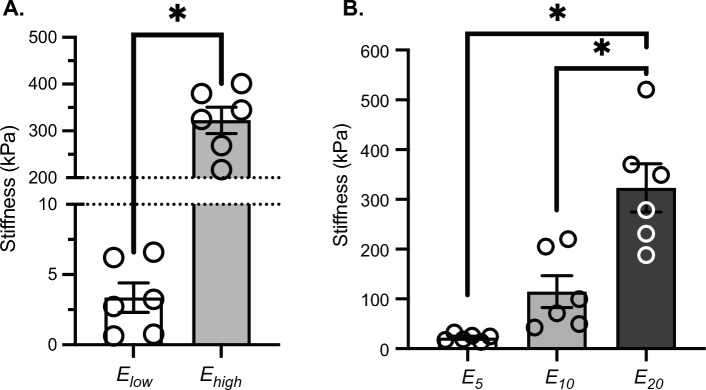
Bladder wall stiffness increases nonlinearly during bladder filling. **A** Average mechanical parameters at low and high pressures during filling. **B** Average mechanical parameters at 5 mmHg (E5), 10 mmHg (E10) and 20 mmHg (E20) during filling. Bladder stiffness significantly increases in a nonlinear fashion as pressure increases during ex vivo bladder filling. * = *p* < 0.05 by paired t test **A** or one-way repeated measures ANOVA (**B**). *N* = 6

Stress is dominated by elastin and uncoiling of collagen fibers early in filling and by the mechanics of the straightened collagen fibers late in filling (Heppner et al. 2016; Tykocki et al. 2019). In the transition region, tissue mechanics are dominated by the microstructure of uncoiling collagen fibers (Zwaans et al. 2022). Biomechanical metrics can be derived from the slope of the linear fit of the stress-stretch relationship early in filling (*E*_low_; low pressure stiffness) and late in filling (*E*_high_; highest pressure stiffness), which quantify the purely elastic behavior of the bladder wall and its constituents. The intersection of the linear fits identified by *E*_low_ and *E*_high_, described by the stretch *λ*_*m*_, represents the mean recruitment stretch of collagen fibers within the bladder wall. When preforming a paired comparison between the values of stiffness evaluated at low and high pressures for each sample, we find that the bladder wall stiffness is significantly greater at high pressures vs. low (Fig. 8).

We also evaluated localized stiffness for three values of pressure: 5 mmHg, 10 mmHg, and 20 mmHg. These three specific values where chosen because they represent three physiologically significant conditions in the micturition cycle, in vivo filling, initiation of voiding, and maximum filling, respectively. When performing a paired comparison, we find that the localized stiffness is significantly increasing at each pressure step we evaluated. This suggests the mechanical environment of the wall is progressively getting stiffer during filling. Taken together, these quantifications strengthen the proposition that a single parameter, such as clinical compliance, is not an accurate representation of the bladder wall mechanics due to the high nonlinearity of the bladder tissue. Instead, measures like those presented here can accurately describe the mechanical behavior throughout the filling process.

## Discussion

Traditionally, bladder compliance was measured experimentally as the change in volume for a given change in infused pressure, due in part to the inability to reliably calculate the volume of the bladder wall without ultrasound imaging (Dorr 1992). Measuring compliance in this manner erroneously makes two key assumptions: first, it assumes that all healthy bladders are the composed of similar proportions of cells and matrix and thus have a similar elastic modulus. Second, it assumes that the total volume and gross geometry of the bladder wall is the same. Because the measures of compliance are also taken at very low pressures as compared to total capacity, “compliance” is easily skewed by the size of the bladder overall. For example, a large bladder with a thin, stiff wall could appear to be highly compliant if only measuring at the lower portion of physiological filling pressures. Also, the mechanical behavior of the bladder wall clearly is nonlinear: intravesical pressure changes very little for very large filling volumes but increases exponentially thereafter (Parekh et al. 2010). To know the relationship between stress, stretch, and bladder filling, new methods are required that accurately measure the volume of the bladder wall itself to calculate stress and compliance. Because the PRIM System allows rapid and accurate measurements of bladder shape, internal volume, and wall volume over the entirety of bladder filling (See *Methods*) while also capturing frame-locked intravesical pressure recordings, compliance can instead be reported as actual engineering metrics (Cauchy stress/stretch) instead of ΔV/ΔP.

Previous work has used similar methods to identify bladder wall mechanical properties in humans in vivo using a combination of cystometry and ultrasound imaging (Dorr 1992; Maddra et al. 2022). These studies highlight the value and importance that this new type of compliance measurement can have with regard to understanding urinary bladder function. In vivo, however, bladder wall geometry was only captured in the sagittal plane due to limitations of ultrasound imaging and sampling rate. This required assumptions to be made with regard to the isotropy (or anisotropy) of the bladder during filling as changes to geometry in the transverse plane were unknown. This technique also has limited applicability to research models of bladder dysfunction due to the highly technical nature of ultrasound imaging, the expense of such devices, and the relative size of rodent bladders as compared to humans. The PRIM System is able to bridge this gap: our system can be used to make similar measurements quickly, accurately, and inexpensively in ex vivo rodent models that can be used to validate the measurements made in humans and the assumptions required therein. Our methods could also determine the ideal plane from which to measure human bladder mechanical properties using the aforementioned techniques. The ex vivo nature of the PRIM System also allows measurements beyond the physiological range of pressures and filling rates, as was conducted herein. Our intention in doing so was threefold: first it allowed us to correlate our mechanical findings to prior literature on ex vivo bladder filling and in vivo cystometry in mice (Heppner et al. 2016). Second, by moving to supraphysiological pressures we could also assess the previously proposed model of collagen fiber recruitment made from uniaxial stress/stretch measurements (Tuttle et al. 2022, 2021) and apply it to the intact bladder configuration. Third, we can now address the interrelationship between ECM, bladder smooth muscle cell structure, and bladder muscle tone/contractility (Fig. 8).

In terms of the calculations of the mechanical properties of the bladder wall, our methods do depart from those used to measure mechanical properties of the bladder in vivo. Previous calculations required that bladder wall thickness be accurately measured at every pressure value during filling (Nagle et al. 2017; Watanabe et al. 1981). Our bladder wall stress calculations consider both the initial thickness and the relative change in radius during filling and not just the surface area of the vessel. Thus, only a single measurement of wall volume is required to accurately calculate stress and stretch at all filling pressures. Due to technical limitations of older imaging techniques, previous investigations also determined the mechanical properties of the whole bladder by assuming that the cross-sectional area of a single plane is representative of all sections of a spherical vessel. While this may be true for some bladders, even a small deviation from the actual central planes can markedly skew the calculated Cauchy stress. With the development of the PRIM System, we are able to accurately measure and calculate the entire wall volume of the bladder without inference; thus, the radius and thickness we use to estimate Cauchy stress better describes the behavior of the entire bladder wall. We also believe this is more representative of the complete mechanical properties of the bladder wall over the entirety of filling and emptying given the marked change in wall thickness that occurs. This allowed us to quantify how the linearized stiffness is significantly different when evaluated for different values of pressure, even when performing a paired comparison for the same samples. This highlights how a single mechanical parameter such as compliance is inadequate to capture the complex mechanical behavior of the bladder wall.

Our calculations, however, are also not without their assumptions that must be considered. We, and others (Nagle et al. 2017; Watanabe et al. 1981), assumed the bladder wall to be a homogeneous material that undergoes and isotropic deformation during filling. The anatomical makeup of the bladder wall is clearly non-homogenous; smooth muscle cells, blood vessels, nerves, and urothelial cells exist heterogeneously throughout the bladder wall (Neuhaus and Schwalenberg 2012). Multiple studies have examined the active and passive behavior of the bladder wall using similar calculations (reviewed in (Roccabianca and Bush 2016)), and it is important to consider that regional differences in mechanical properties do occur (Tuttle et al. 2021). Nonetheless, given the paucity of information as the biomechanical properties of the intact bladder wall during filling, the value of the measurements included here are not diminished and serve as a springboard for more in-depth measurements in the future. We also assume the bladder to be a spherical vessel undergoing isotropic deformation during filling. The first of these assumptions, sphericity, was made for simplicity: in this initial study, we were less curious as to the specific stresses relative to the angular deformation and more curious as to the average stresses experienced at any single point within the bladder wall at a given moment. While the spherical assumption made for the stress analysis is an approximation, it is a necessary one. This is due to the lack of automatized quantification of the geometry throughout the filling test, having manual quantification of all diameters would introduce an operator-dependent factor that the current analysis minimizes. Furthermore, the spherical assumption has been consistently used in the past when modeling the urinary bladder (Nagle et al. 2017; Regnier et al. 1983; van Mastrigt and Griffiths 1986; Watanabe et al. 1981). Given the data collected, we are able to calculate stretch and stress as it varies with any polar angle during filling as done previously (Damaser and Lehman 1995) and intend to do so in future studies. The second assumption, isotropy, is yet another current limitation we can overcome in future studies given the data we collect in the PRIM System. The bladder wall mechanical properties are most often measured using uniaxial testing paradigms best suited for isotropic materials, even though the wall itself is known to be anisotropic (Ajalloueian et al. 2018). The results presented here are intended to serve as a comparator by which our methodologies and results can be related to those gleaned from uniaxial testing measurements. Because our samples are completely intact during the entirely of filling and five image planes are available for each data point, assessment of both anisotropy and angular deformation during filling is obtainable.

In conclusion, we developed the PRIM System in order to measure the mechanical properties of the bladder wall over the entirety of bladder filling since the information derived from pressure–volume curves is incomplete. Using this relatively simple imaging technique, we are able to combine measurements of intravesical pressure and infused volume with wall geometry and wall thickness to calculate Cauchy stress and stretch during ex vivo bladder filling. Thus, the PRIM system offers unprecedented insights into the relationship between stress, stretch, and contractility that govern normal bladder function. Further, this technology can be used to measure the same properties in other hollow organs, such as uterus, gut, and stomach, and also be used to generate dynamic four-dimensional models of changes in shape of these organs with high temporal resolution. The PRIM System is thus a promising new tool for understanding how muscle contractions coordinate across an entire organ and the mechanical forces that accompany those contractions.

### Supplementary Information

Below is the link to the electronic supplementary material.

Supplementary file1 (DOCX 17 KB)

Supplementary file2 (GIF 65360 KB)
