# Supplementary material for: Quantifying whole bladder biomechanics using the novel pentaplanar reflected image macroscopy system
Source: Biomech Model Mechanobiol. 2023 May 30;22(5):1685–95. doi: 10.1007/s10237-023-01727-0 (PMC10511590; doi:10.1007/s10237-023-01727-0)
Supplement: Supplementary file 1 — Supplementary file1 (DOCX 17 KB) [file 10237_2023_1727_MOESM1_ESM.docx]

**SUPPLEMENTARY VIDEO LEGEND**

**Video 1** Bladder filling in the PRIM System. Mouse bladder ex vivo filling as pressure increases from 0 to 20 mmHg. Frame rate is increased 8-fold.
