# Supplementary figures and images for: Quantifying whole bladder biomechanics using the novel pentaplanar reflected image macroscopy system
Source: Biomech Model Mechanobiol. 2023 May 30;22(5):1685–95. doi: 10.1007/s10237-023-01727-0 (PMC10511590; doi:10.1007/s10237-023-01727-0)

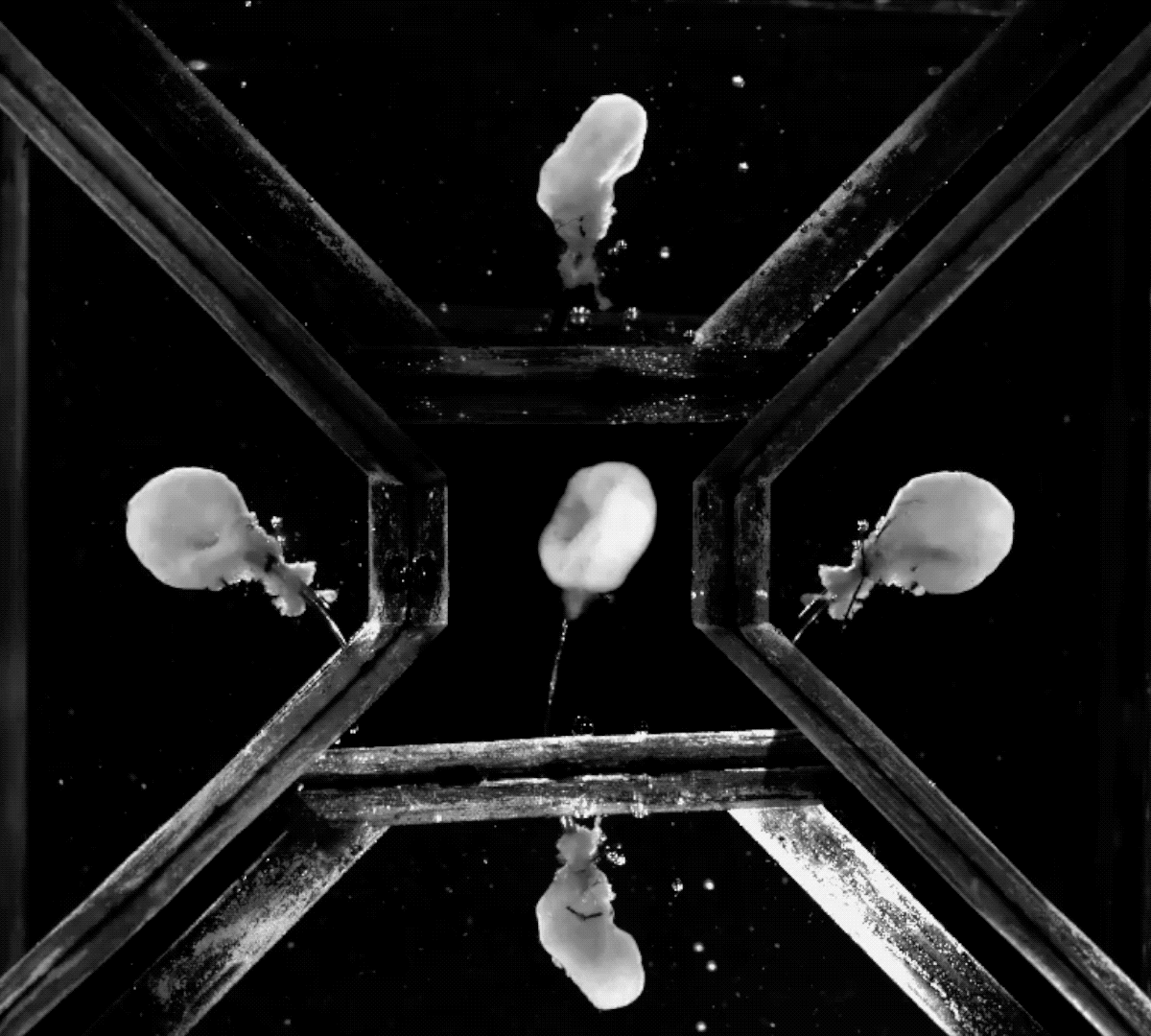

Supplement: Supplementary file 2 — Supplementary file2 (GIF 65360 KB) [file 10237_2023_1727_MOESM2_ESM.gif]
